# Preliminary feasibility of integrating tobacco treatment into SUD peer recovery coaching: a mixed-methods study of peer recovery coaches

**DOI:** 10.1186/s13722-023-00380-3

**Published:** 2023-04-30

**Authors:** Joanna M. Streck, Susan Regan, Michael Werner, Alexia Glynn, Andrea C. Villanti, Elyse R. Park, Sarah E. Wakeman, A. Eden Evins, Nancy A. Rigotti

**Affiliations:** 1 Department of Psychiatry, Massachusetts General Hospital (MGH), 100 Cambridge Street, 16th Floor, Boston, MA 02114 USA; 2 Harvard Medical School, Harvard University, Boston, MA USA; 3 Tobacco Research and Treatment Center, Division of General Internal Medicine, Department of Medicine, MGH, Boston, MA USA; 4 Rutgers Center for Tobacco Studies, Rutgers University, New Brunswick, NJ USA

**Keywords:** Substance use disorder, Peer recovery coach, Tobacco, Cigarette smoking

## Abstract

**Background:**

Individuals with substance use disorder (SUD) have high prevalence of cigarette smoking and difficulty quitting. Peer recovery coaches (PRCs; individuals with lived SUD experience) facilitate SUD behavior change in recoverees but it is unknown if/how they address tobacco treatment in SUD recovery coaching. We assessed PRC’s tobacco-related practices and attitudes about tobacco treatment in SUD recovery.

**Methods:**

The Tobacco use In Peer-recovery Study (TIPS) was a cross-sectional mixed-methods pilot survey (January–March 2022) of the 26 PRCs employed by a Massachusetts-based healthcare system’s 12 SUD treatment clinics/programs. PRCs completed a quantitative survey (n = 23/26; 88%) and a telephone-based qualitative interview (n = 20/26; 77%).

**Results:**

One-third of PRCs reported current smoking, 50% reported former smoking, and 18% never smoked. Among PRCs, 61% reported accompanying recoverees outdoors to smoke, 26% smoked with recoverees, 17% had provided cigarettes to recoverees, 32% used smoking to help build peer-relationships, and 74% rated smoking as socially acceptable in SUD treatment. PRCs reported regularly talking to recoverees about tobacco treatment (65%), believed they should have a role in helping recoverees quit smoking (52%), and were interested in tobacco treatment training (65%). A majority of both nonsmoking and current smoking PRCs (73% vs. 57%) regularly talked to recoverees about quitting smoking.

**Conclusion:**

PRCs’ attitudes about integrating tobacco treatment into SUD recovery coaching were generally positive and PRCs reported they could have a role in helping recoverees with tobacco treatment. Barriers to integrating tobacco treatment into SUD recovery include use of cigarettes as a peer-recovery tool and high prevalence and social acceptability of smoking in SUD recovery.

**Supplementary Information:**

The online version contains supplementary material available at 10.1186/s13722-023-00380-3.

## Background

Tobacco smoking is responsible for nearly 500,000 deaths each year [1]. Despite promising declines in smoking prevalence in the general population across the past decade, as well as among individuals with substance use disorder (SUD), disparities in tobacco use persist [2]. In the U.S., individuals with SUD have nearly a three-fold higher prevalence of cigarette smoking compared to individuals without SUD (i.e., 36% vs. 13%, respectively)[2, 3]. There is a growing consensus that even with effective evidence-based tobacco use disorder (TUD) treatment (i.e., FDA-approved medications and counseling), smoking cessation rates for those with SUD remain modest and lower than the quit rates among individuals without SUD [4–6].

There are several treatment-systems and social factors that likely contribute to the low rates of smoking cessation among individuals with SUD. First, national estimates suggest that while the number of SUD treatment clinics offering smoking cessation services has increased over time, this practice is still generally uncommon with < 50% of SUD clinics offering smoking cessation counseling for example.[7, 8]. Additionally, there have been widely documented misconceptions of tobacco use by SUD and other mental health professionals including inaccurate perceptions that people with SUD don’t want to quit tobacco, that quitting smoking is “too much” for patients to take on, and the perception that cigarettes can be a useful tool for SUD patients and staff [9].

Other major social factors which likely inhibit TUD interventions among people with SUD, and factors that have not been targeted in existing TUD interventions, are the high rates of current tobacco use in SUD treatment, among both SUD recoverees (i.e., individuals in SUD recovery) and clinic staff, the social acceptability of smoking, and the limited social support for quitting [5, 10, 11]. The high prevalence of cigarette smoking among staff members at SUD treatment facilities is associated with increased smoking in SUD recoverees and decreased receipt of tobacco cessation by recoverees [12]. Innovative tobacco cessation treatments for individuals in SUD treatment are urgently needed, including interventions that address social factors related to smoking and SUD treatment-system components that help sustain smoking in this population. Ensuring TUD efforts in SUD treatment facilitate engagement in SUD care, do not serve as a barrier to SUD treatment entry or engagement, and are patient-centered are crucial considerations, as is the importance of offering equitable TUD services to all patients [9].

Peer-recovery coaches (PRCs) are individuals with lived SUD experience (in SUD recovery themselves), who provide peer-support coaching to recoverees in SUD treatment [13]. Converging data suggests that PRC contact is associated with positive SUD behavior change including increased treatment attendance, abstinence rates, decreased rates of recurrence of substance use and decreased stigma. Recoverees report satisfaction with PRC support and describe peer coaching as helpful for promoting behavior change [13–15]. SUD PRCs could potentially be leveraged to assist with tobacco treatment to help individuals with SUD quit smoking. However, no work has examined SUD PRC’s tobacco smoking rates or reported on their attitudes regarding integrating tobacco treatment coaching into their SUD recovery coaching. Addressing these scientific gaps can inform the development of tobacco treatment interventions for those with SUD, including the feasibility and acceptability of peer delivered TUD interventions.

Helping recoverees in SUD treatment quit smoking is an urgent priority especially as cigarette smoking is associated with increased SUD relapse, and smoking cessation has been found to improve SUD treatment outcomes, in addition to reducing tobacco-related morbidity and mortality [16–18]. The aims of the present investigation were to investigate SUD PRC’s (1) current cigarette smoking status, (2) current tobacco-related practices with their SUD recoverees, and (3) attitudes about integrating tobacco treatment into SUD recovery generally and into their SUD coaching sessions more specifically. The present cross-sectional investigation represents an exploratory, hypothesis-generating survey study assessing behaviors and attitudes of SUD PRCs and a preliminary assessment of the feasibility of PRCs supporting cessation efforts in their SUD recoverees [19].

## Methods

### Design and participants

The Tobacco use In Peer recovery Study (TIPS) was a mixed-methods, cross-sectional pilot study of SUD PRCs employed by a large Massachusetts-based healthcare system (12 SUD treatment programs) conducted January-March 2022. All SUD PRCs employed by the Mass General Brigham (MGB) healthcare system (N = 26) were sent an email containing a link to a confidential quantitative survey self-administered by the PRC in REDCap. Those who completed the survey were offered participation in an individual telephone-based qualitative interview which occurred after quantitative survey completion to allow the qualitative interview guide to be informed by the quantitative survey responses consistent with an explanatory sequential mixed methods design. Participants were remunerated $50 for completion of the quantitative survey and an additional $50 for completing the qualitative interview. All study procedures were approved by the Mass General Brigham (MGB) institutional review board and the internal MGB Recovery Coach Collaborative network (RCC; which includes MGB SUD leadership and PRC managers and leaders across the healthcare system). The quantitative survey items and qualitative interview domains were informed by the empirical literature and iteratively developed and revised in consultation with the MGB RCC and two PRC employees at MGB.

### Quantitative survey measures

#### Demographic and smoking characteristics of SUD peer recovery coaches (PRCs)

We assessed age, gender, race/ethnicity, and education level of PRCs, using items previously validated by our research group in this population [20, 21], other PRC characteristics including whether PRCs completed the state PRC training certification (yes/no), number of years worked as a SUD PRC, and SUD the PRCs were in recovery from [22]e.g., alcohol, cocaine, opioids, tobacco). Participants were asked about ever (smoked 100 cigarettes in lifetime; yes/no) and current (past 30-day) use of cigarettes and non-cigarette tobacco products (e.g., e-cigarettes, cigars) [20, 21]. Those who endorsed past 30-day cigarette smoking were queried about their number of cigarettes smoked per day in the past 30 days, interest in quitting smoking (on a 1–10 scale with 1 = not at all interested and 10 = extremely interested) [23], and history of a past-year quit attempts (≥ 24 h of nonsmoking with intention to quit; yes/no)[24].

#### PRC’s tobacco-related practices and tobacco treatment attitudes

PRCs were asked about their current tobacco-related practices with their SUD recoverees. In this series of items, we assessed the frequency with which PRCs smoked with their recoverees, accompanied recoverees outside from a healthcare facility where that time together included the recoveree smoking, provided recoverees with cigarettes to smoke, used cigarette smoking to build relationships, asked recoverees about their tobacco use, talked with recoverees about their desire to quit smoking/concerns about smoking, provided advice or suggestions for quitting or reducing cigarette use, and recommended use of e-cigarettes instead of cigarettes to reduce harms from tobacco. All responses were ranked on a 4-point Likert scale of frequency ranging from 0 “never” to 4 “always.”

We assessed PRC’s commitment to tobacco treatment using the previously validated Tobacco Treatment Commitment Scale (TTCS), a measure also shown to be associated with the quality of tobacco treatment services in SUD treatment facilities [11, 25]. We used 7 of the original 14 TTCS items selecting items from all 3 factors previously validated in a confirmatory factor analysis (i.e., “tobacco is less harmful than other drugs;” “it’s not our job to treat tobacco,” and “tobacco treatment will harm clients”). Each item was rated on a 5-point Likert scale ranging from 1 ”strongly agree” to 5 ”strongly disagree,”[11], with additional anchors of 2 “agree”, 3 ”neither agree or disagree”, and 4”disagree” added by our research group.

Using the same 5-point Likert scale,[11, 25] we asked PRCs additional questions pertaining to their attitudes about integrating tobacco treatment into their SUD recovery coaching (i.e., *“*Peer recovery coaches should have a role in helping individuals in SUD treatment quit smoking” and *“*I am interested in receiving additional training on how to help recoverees cut back or quit smoking”). We also assessed PRC’s perceptions of the social acceptability of smoking cigarettes in SUD treatment using an established measure of social acceptability of smoking (i.e., “among your recoverees, how socially acceptable do you think cigarette smoking is?”) with response options ranging from 1 “not at all socially acceptable” to 5 “extremely socially acceptable”[26, 27].

### Qualitative interview

All survey participants who indicated interest in completing a qualitative interview were called by research staff to complete a 30–60 min individual telephone interview that was audio-recorded and transcribed for analysis. All qualitative interviews were administered by a trained research staff member (MW) who completed a 2-hour institutional qualitative interviewing training through the MGH Qualitative and Mixed Methods Research Unit and supervision sessions led by the study PI (JMS) and the PI’s qualitative mentor (ERP). Using a semi-structured interview guide, staff investigated the following qualitative interview domains: PRC’s current tobacco-related coaching practices, PRC’s attitudes about their role in tobacco treatment, PRC’s attitudes about the priority of tobacco treatment in SUD peer coaching, and thoughts on how PRC’s own cigarette smoking may help or harm their ability to help a recoveree quit smoking. The qualitative interview guide was informed and tailored by the quantitative survey data which research staff reviewed for each participant prior to administering the qualitative interview. The qualitative data clarified the *reasons* for current tobacco-related practices by coaches, perceptions, and the level of intensity of beliefs. For example, when asking PRCs about current tobacco-related practices with recoverees, the qualitative interview guide prompted research staff to review the participant’s response from the quantitative survey (i.e., “You mentioned on our last survey, that [insert answer to quantitative survey item on current tobacco practice (s)]. Could you please tell me more about this? Can you tell me why you did this?”).

### Statistical methods

Descriptive statistics were used to characterize PRC demographic and smoking characteristics. Quantitative survey questions on PRC tobacco-related practices and tobacco treatment attitudes were explored by PRC’s past 30-day cigarette smoking status (yes/no) using chi-squared tests and t-tests. Likert scales for the PRC tobacco-related practices items were dichotomized (i.e., never/rarely vs. sometimes/usually/always) and scales for the tobacco attitudes questions were also collapsed (i.e., strongly agree/agree, neither agree nor disagree, strongly disagree/disagree. Quantitative analyses were conducted in STATA version 16 (StataCorp. 2019, College Station, TX: StataCorp LLC), with statistical significance set at p < 0.05.

Qualitative interviews were iteratively coded by four study team members (MW, SG, AG, JS) trained in qualitative analysis (trained and supervised by ERP) using the constant comparative method [28]. Coders reviewed five transcripts to develop coding reliability and a preliminary codebook. Subsequent transcripts were coded and reviewed weekly, and discrepancies were resolved in meetings by consensus with the project PI (JMS). Once thematic saturation was reached, relevant themes/subthemes were extracted and compared by PRC cigarette smoking status (i.e., current vs. former vs. never smoking). The quantitative data was analyzed first to inform qualitative analyses consistent with an explanatory sequential mixed methods design [29].

## Results

### Survey response

Of the 26 SUD PRCs employed by the MGB healthcare system across 12 SUD treatment clinics/programs, 23 (88%) completed the quantitative survey and 20 (77%) completed the individual qualitative interview.

### Peer recovery coach characteristics

PRC demographic and smoking characteristics are presented in Table 1. PRCs mean age was 51 years, 57% were male, 65% identified as white, and their mean tenure as a PRC was 7 years. Nearly one-third (n = 7; 32%) of PRCs reported current past 30d cigarette smoking, 50% (n = 11) reported former smoking (i.e., endorsed ever smoking but no past 30d smoking), 18% (n = 4) had never smoked cigarettes, and one coach had an unknown smoking status. Some PRCs (n = 5, 22%) reported past 30-day use of non-cigarette tobacco products (e.g., e-cigarettes) (Table 1).

**Table 1:** Demographic and smoking characteristics of SUD peer recovery coaches enrolled in the TIPS Study (N = 23)

	N (%) or Mean (SD)
Demographic characteristics	
Age	51 ± 11
Gender	
Male	13/23 (57%)
Female	10/23 (43%)
Race	
White	17/23 (74%)
Black or African-American	4/23 (17%)
Other	2/23 (9%)
Ethnicity	
Hispanic	2/23 (9%)
Non-Hispanic	20/23 (87%)
Don’t know	1/23 (4%)
Education	
HS graduate or equivalent	5/23 (22%)
Some college	10/23 (43%)
2- or 4- year college degree	8/23 (35%)
PRC characteristics	
Obtained peer recovery coach state certification	15/23 (65%)
Other certifications (e.g., licensed drug/alcohol counselor)	11/23 (48%)^a^
Years worked as PRC	7 ± 8
Number of SUDs from which PRC was in recovery from	3 ± 3
SUDs from which PRC was in recovery from	
Alcohol	20/23 (87%)
Cannabis	10/23 (43%)
Cocaine	13/23 (57%)
Prescription stimulants	1/23 (4%)
Methamphetamine	3/23 (13%)
Tobacco cigarettes	12/23 (52%)
Inhalants	1/23 (4%)
Sedatives	6/23 (26%)
Hallucinogens	3/23 (13%)
Street opioids	10/23 (43%)
Prescription opioids	6/23 (26%)
Other	2/23 (9%)
Prefer not to answer	1/23 (4%)
Tobacco use characteristics	
Smoking status^b^	
Never smoking	4/22 (18%)
Former smoking	11/22 (50%)
Current (past 30d) smoking	7/22 (32%)
Cigs/day in past 30 (among past 30d smokers)	11 ± 7
Quit interest (among past 30d smokers)^c^	8 ± 3
Prior lifetime quit attempt (among ever smokers)	14/17 (82%)
Past 30d alternative tobacco product use^d^	
E-cigarettes	2/23 (9%)
Smokeless tobacco	1/23 (4%)
Cigars/Pipes/Hookah/Cloves/bidis/snus/paan with tobacco	0/23 (0%)
Other	1/23 (4%)

Key: Yrs, years; d, day; SUD, substance use disorder; PRC, peer recovery coach

^a^BHJI navigator, LADC, certified peer specialist, trauma specialist, reiki certificate

^b^1 participant had unknown past 30d smoking status and is not included

^c^Quit interest was a single item with responses ranging from 1 = not at all interested to 10 = extremely interested

^d^Of the n = 2 e-cigarette users, n = 1 was a current cigarette smoker and the other person had a missing past 30d smoking status. Of the n = 1 smokeless tobacco user, the participant was a non-cigarette smoker in the past 30d. Of the n = 1 who endorsed “other” past 30d tobacco products, the participant endorsed use of “zin pouches” (this participant was a non-cigarette smoker in the past 30d)

### Tobacco-related peer coaching practices

Among all PRCs, 61% reported accompanying recoverees outside to smoke, 26% had smoked with recoverees, 17% had provided cigarettes to recoverees, and 32% used smoking to help build peer-relationships (Table 2). Most PRCs also reported regularly asking recoverees about their cigarette smoking (55%), talking to recoverees about tobacco treatment (65%), and advising recoverees to quit smoking (60%). No PRC reported tobacco-related practices with recoverees differed by PRCs’ past 30-day smoking status other than PRCs who currently smoke being more likely to smoke with recoverees (Table 2).

**Table 2:** Tobacco-related peer coaching practices and attitudes by SUD peer coaches past 30-day cigarette smoking status

N (%) Tobacco-related coaching practices	All (N = 23)	Not smoking (n = 15/22; 68%)	Smoking (n = 7/22; 32%)	P value
*Pro-smoking practices*				
Smoke with recoverees				**< 0.001**
Never/rarely	17/23 (74%)	14/15 (93%)	2/7 (29%)	
Sometimes/usually/always	6/23 (26%)	1/15 (7%)	5/7 (71%)	
Accompany recoverees out to smoke				0.08
Never/rarely	9/23 (39%)	8/15 (53%)	1/7 (14%)	
Sometimes/usually/always	14/23 (61%)	7/15 (47%)	6/7 (86%)	
Give cigarettes to recoverees				0.39
Never/rarely	19/23 (83%)	13/15 (87%)	5/7 (71%)	
Sometimes/usually/always	4/23 (17%)	2/15 (13%)	2/7 (29%)	
Use cigarette smoking to build relationship with recoverees^a^				0.51
Never/rarely	15/22 (68%)	10/14 (71%)	4/7 (57%)	
Sometimes/usually/always	7/22 (32%)	4/14 (29%)	3/7 (43%)	
*Pro-Cessation Practices*				
Ask recoverees about their smoking^b^				0.68
Never/rarely	10/22 (45%)	6/15 (40%)	3/6 (50%)	
Sometimes/usually/always	12/22 (55%)	9/15 (60%)	3/6 (50%)	
Talk to recoverees about quitting				0.45
Never/rarely	8/23 (35%)	4/15 (27%)	3/7 (43%)	
Sometimes/usually/always	15/23 (65%)	11/15 (73%)	4/7 (57%)	
Advise recoverees to quit^c^				0.83
Never/rarely	8/20 (40%)	5/13 (38%)	2/6 (33%)	
Sometimes/usually/always	12/20 (60%)	8/13 (62%)	4/6 (67%)	
Recommend e-cigarettes to reduce cigarette-related harms^d^				0.31
Never/rarely	15/22 (68%)	11/15 (73%)	3/6 (50%)	
Sometimes/usually/always	7/22 (32%)	4/15 (27%)	3/6 (50%)	
Attitudes about Tobacco Treatment in SUD Treatment				
*Social acceptability of cigarette smoking among recoverees*				0.46
Not at all/somewhat unacceptable	2/23 (9%)	2/15 (13%)	0/7 (0%)	
Neutral	4/23 (17%)	2/5 (13%)	2/7 (29%)	
Somewhat/extremely acceptable	17/23 (74%)	11/15 (73%)	5/7 (71%)	
*PRCs should have a role in helping individuals in SUD treatment quit smoking* ^a^				0.79
Strongly agree/agree	12/23 (52%)	8/14 (57%)	3/7 (43%)	
Neither agree nor disagree	7/23 (30%)	4/14 (29%)	3/7 (43%)	
Strongly disagree/disagree	3/23 (13%)	1/14 (7%)	1/7 (14%)	
*Interest in additional training on how to help recoverees quit smoking*				0.07
Strongly agree/agree	15/23 (65%)	9/15 (60%)	6/7 (86%)	
Neither agree nor disagree	7/23 (30%)	6/15 (40%)	0/7 (0%)	
Strongly disagree/disagree	1/23 (4%)	0/14 (0%)	1/7 (14%)	

Cells represent N (%) with column % listed for past 30d smoking status cells. P values are from chi-squared tests. For past 30-day smoking status, 1 participant had unknown past 30d smoking status, thus their data is not presented. n = 10/14 of the PRCs who reported nonsmoking in the past 30d reported formerly smoking

^a^n=1 indicated prefer not to answer and their data is not included

^b^n=1 indicated prefer not to answer and their data is not included

^c^n=2 indicated prefer not to answer and n = 1 indicated don’t know and their data is not included

^d^N=1 indicated prefer not to answer and their data is not included

### PRC commitment to and attitudes about tobacco treatment

Most PRCs believed they should have a role in helping recoverees cut back or quit smoking (52%) and were interested in receiving training on delivering tobacco treatment (65%). More nonsmoking PRCs held the belief that they should have a role in helping recoverees quit smoking compared to current smoking PRCs (Table 2). However, 74% of all PRCs rated smoking as highly socially acceptable in SUD treatment. PRCs’ self-reported commitment to tobacco treatment (i.e., the TTCS) are presented in Table 3. PRCs generally disagreed with the two survey items that corresponded to the factor “Tobacco is less harmful than other drugs” (mean of both items = 3.5/5), with more nonsmoking vs. current smoking PRCs expressing disagreement with this statement. However, all PRCs were neutral regarding whether tobacco treatment should be part of the overall mission of SUD treatment programs (mean 2.9). Nonsmoking PRCs generally reported higher mean levels of commitment to tobacco treatment in SUD recovery compared to current smoking PRCs (Table 3).

**Table 3:** Tobacco Treatment Commitment Scale (TTCS) ratings among SUD peer recovery coaches by coaches smoking status

Mean (standard deviation)		Past 30d Smoking Status^a^		
*All items scored on a Likert scale ranging from 1 = strongly agree to 5 = strongly disagree*	All (N = 23)	Not Smoking (n = 15/22; 68%)	Smoking (n = 7/22; 32%)	P value
Factor 1. “Tobacco is less harmful than other drugs”				
Tobacco is less harmful than other SUDs, M (SD)	3.7 (1.1)	4.2 (0.80)	2.6 (0.98)	**< 0.001**
Tobacco causes few problems for my recoverees, M (SD)	3.4 (1.1)	3.7 (0.91)	2.9 (1.2)	0.08
Factor 2. “It’s not our job to treat tobacco”				
Treating tobacco dependence *should* be part of the mission of SUD treatment programs, M (SD)	2.9 (1.1)	2.9 (1.18)	3.0 (1.15)	0.89
SUD treatment programs should not treat tobacco because it isn’t what clients are in treatment for, M (SD)	3.2 (1.1)	3.2 (1.1)	3.1 (1.1)	0.91
Factor 3. “Tobacco treatment will harm clients”				
Treating tobacco dependence will interfere with a recoveree’s other SUD recovery, M (SD)	3.3 (1.1)	3.6 (1.0)	2.7 (1.3)	0.08
Smoking helps recoverees cope with the stress in their lives, M (SD)	2.4 (0.79)	2.4 (0.84)	2.3 (0.76)	0.85
It’s unfair to take recoverees tobacco away from them, M (SD)	2.1 (0.99)	2.3 (1.0)	1.9 (1.1)	0.37

Note. Cells depicting mean (standard deviation) excluding those who reported “don’t know.” P values based on t-tests. The TTCS is recommended to be scored on a 1–5 scale ranging from 1”strongly agree” to 5 “strongly disagree.[11, 25]” Our study included additional qualitative anchors of 2 “agree”, 3 ”neither agree or disagree”, 4”disagree.”

^a^1 participant had unknown past 30d smoking status and was not included

### Qualitative data

Qualitative themes and representative quotes are summarized in Additional file 1: Table S1.

A common theme across all qualitative domains and PRC smoking statuses was the importance of tobacco coaching in a patient-centered manner. Specifically, PRCs emphasized that they generally were supportive of addressing tobacco treatment in SUD coaching, but only *if* a recoveree expressed interested in coaching and listed TUD as a coaching goal.

When examining the data by PRC smoking status, most former smoking PRCs were supportive of addressing tobacco cessation in SUD treatment and in SUD peer coaching. PRCs identifying as former (vs. current) smokers described that treating tobacco was a high priority in SUD coaching and several coaches noted that they were already coaching recoverees on quitting using both their lived-experience and behavioral skills (e.g., identify and distract from triggers for smoking cigarettes). Former smoking coaches believed that they should serve as a model of nonsmoking for recoverees and help guide them through cessation based on their own journey quitting smoking. A few coaches reported they were certified tobacco treatment specialists and were implementing behavioral skills for tobacco cessation.

However, many current smoking PRCs explained that smoking cessation was a low priority during their coaching and shared that they infrequently discussed smoking with their recoverees. This viewpoint was also held by a few former and never smoking PRCs. This viewpoint was explained to be largely due to PRC’s lack of training in treating tobacco, the perception that they were not experts in smoking cessation treatment, the perception that tobacco wasn’t a patient’s coaching goal, and/or the belief that tobacco is less of an immediate health threat compared to other substances (e.g., the potential for imminent risk of overdose with use of unregulated opioids).

## Discussion

We conducted a pilot mixed-methods, cross-sectional study of SUD PRCs employed by a large Massachusetts-based healthcare system’s 12 SUD treatment clinics/programs (88% survey response rate). We examined PRC’s smoking status, their tobacco-related practices with recoverees, and their attitudes about integrating tobacco treatment in their SUD coaching.

The PRC cigarette smoking status observed in our study is generally comparable to the smoking status reported by Guydish and colleagues [12] in their survey in 2019–2022 of California residential SUD treatment program staff (any full or part time staff). Specifically, Guydish and colleagues reported that 22% of staff reported current (at time of survey) smoking and 49% reported former smoking. The higher rate of current smoking by PRCs in the present study (i.e., 32%) is likely attributable to the fact that our sample represented staff in recovery from SUD. The prevalence of cigarette smoking among PRCs in the present study and that among SUD clinic staff in the prior study are lower than that observed among patients enrolled in SUD treatment (60% of the SUD treatment clients in the Guydish study reported current cigarette smoking). Nonetheless, rates of smoking in clinic staff and PRCs are higher than rates seen in the general population (~ 13% smoking prevalence nationally [30].

Overall, the survey responses indicate that peer coaches view tobacco treatment as an important goal to be supported when identified by the patient. This is congruent with the overall framework of the recovery coaching model [14, 31], which is based on supporting the recoveree’s self-identified goals, rather than a coach or clinician imposing goals or specific treatments. Importantly, PRCs viewed tobacco as harmful and were interested in learning more about how to support tobacco cessation in SUD recovery. Similar to prior studies among SUD and mental health staff and providers, PRCs identified that smoking was generally seen as socially acceptable in SUD treatment [9, 11, 12]. Given the high prevalence of tobacco use among people with SUD, it is not unexpected that PRCs would be with recoverees who were smoking during that encounter. The PRC role is designed to be focused on engagement and PRCs are uniquely able to connect with participants in non-traditional settings, for example meeting them in community-based settings which allows them to engage in ways that clinical care team members are not able to. Nonetheless, some PRCs reported providing recoverees with cigarettes and/or smoking with recoverees when outside together to build the relationship which could be a barrier to tobacco treatment implementation and suggests a need for PRC training in tobacco use and treatment prior to tobacco treatment delivery.

Most PRCs also believed they should have a role in helping recoverees quit smoking, especially those PRCs who had successfully quit smoking themselves. Similarly, both our quantitative and qualitative data revealed that nonsmoking PRCs (comprised mostly of former smoking coaches) were more engaged and committed to tobacco treatment compared to current smoking PRCs. Prior studies examining cigarette smoking by SUD clinic staff have found that SUD clinic staff (comprised mostly of administrators and leaders such as clinic directors) who smoke were less likely to endorse treating clients’ TUD [11, 12] and this appears to be true with PRCs who currently smoke as well.

On the TTCS, while most PRCs disagreed with the misconception that tobacco is less harmful than other SUDs, both current and nonsmoking PRCs expressed neutrality about whether tobacco treatment should be part of the general mission of SUD treatment programs. This viewpoint was explained in the qualitative data with most coaches reporting that tobacco treatment should only be part of the mission of SUD treatment for recoverees who express interest in tobacco treatment, but it shouldn’t be forced on anyone or recommended unless a recoveree specifically expresses a desire to quit and to receive help from a coach, consistent with the general philosophy of recovery coaching described above. PRCs continually expressed throughout the interviews that their coaching practices are guided by the primary tenant of being patient centered. This framework aligns with a widely recognized approach to SUD treatment recommended to strengthen the quality of treatment (i.e., patient-centered care)[32, 33]. This is a contrast to the smoking cessation guidelines for clinical healthcare providers that recommend all individuals who smoke cigarettes, regardless of their quit interest, be offered evidence-based smoking cessation services and may represent an important philosophical difference that traditional TUD approaches need to contend with in order to better integrate into SUD care [34].

Our study was limited in that we did not survey PRCs outside of the MGB healthcare system thus our results do not generalize beyond one Massachusetts healthcare system. However, our healthcare system is large and includes 12 diverse SUD treatment clinics/programs (with 1–2 PRCs employed per clinic). Given the total sample size of coaches across our healthcare system, we were unable to plan/conduct multivariate testing and we were likely underpowered to detect differences by smoking status. Thus, future work using larger sample sizes could more adequately assess how PRC attitudes about tobacco treatment differ across PRC smoking status. However, our findings are strengthened by a mixed-methods design with a robust qualitative sample size of n = 20 which allowed for thematic saturation to be reached. Additionally, we did not assess PRC’s knowledge of or experience with smoking cessation pharmacotherapies which is an important future research direction that could inform treatment development efforts. Finally, we did not biochemically-confirm tobacco smoking status and thus our findings are limited to PRC self-report which could be subject to social desirability, though our survey was confidential and self-administered by the PRC.

To our knowledge, this is the first exploration of smoking status and attitudes towards tobacco treatment among SUD peer recovery coaches. We found a lower prevalence of smoking by PRCs compared to recoverees/patients in SUD treatment, and generally favorable attitudes towards integrating tobacco treatment into SUD recovery coaching, with more favorable attitudes towards treatment held by former vs. current smoking coaches. It may be feasible for former smoking PRCs to deliver tobacco treatment interventions to a population in urgent need of novel tobacco treatment methods. However, this approach warrants scientific testing. Barriers to integrating tobacco coaching into SUD treatment remain and include the use of cigarettes as a peer recovery tool and the need to adapt TUD approaches to fit within a PRC’s patient-centered coaching framework and existing SUD treatment frameworks (e.g., harm reduction).

## Supplementary Information

**Additional file 1:** **TableS1.** Qualitative themes identified by peer recovery coaches on integrating tobacco treatment into SUD recovery coaching.

## Data Availability

Data and materials will be available upon reasonable request.
