# Supplementary material for: Preliminary feasibility of integrating tobacco treatment into SUD peer recovery coaching: a mixed-methods study of peer recovery coaches
Source: Addict Sci Clin Pract. 2023 Apr 30;18:25. doi: 10.1186/s13722-023-00380-3 (PMC10148997; doi:10.1186/s13722-023-00380-3)
Supplement: Supplementary file 1 — Additional file 1: TableS1. Qualitative themes identified by peer recovery coaches on integrating tobacco treatment into SUD recovery coaching. [file 13722_2023_380_MOESM1_ESM.docx]

Supplemental Table 1. Qualitative themes identified by peer recovery coaches (PRCs) on integrating tobacco treatment into SUD recovery coaching (N=20)

| **Interview Domain** | **Identified Theme** (including smoking status of PRCs most often endorsing theme) | **Definition of Theme or Sub-themes** | **Representative Quote(s)** |
| --- | --- | --- | --- |
| **Current tobacco-related practices during SUD coaching** |  |  |  |
|  | Current smoking PRCs: Smoking doesn't come up in my work with recoverees in SUD treatment | - Tobacco treatment is not a stated coaching goal for most patients - PRC not trained on tobacco treatment so don’t address tobacco treatment with recoverees | *“Yep. Well, I mean never in the sense that I wouldn't bring it up. I mean, I think that if they brought it up I would send them to someone to talk about it.” (current smoking PRC)* |
| Pro-cessation Practices | | | |
|  | All PRCs: Ask recoverees about their smoking | - Bring up smoking casually in conversation - Ask recoverees how smoking is related to their other SUD use | *“I just simply ask a question - have you ever thought about it? - or when we do talk about it [smoking], we talk about the actual price. You know what I mean? How expensive it is to be a smoker these days. Yeah. I mean, that's pretty much it.” (current smoking PRC)*  *“I ask them how much are they are smoking and why do they smoke, what's causing them to smoke. Are they aware they're smoking? Because sometimes I know I used to smoke a cigarette, and I'd say, "But I just smoked a cigarette, and I've got another one. Why?" And I didn't even notice I lit up another cigarette back-to-back. And that was not always the case, and there's patterns. Either [I was using?] a different drug or I had anger or I was nervous. And the thing is about importance of being aware why one is smoking.”*  *(former smoking PRC)* |
|  | Former/never smoking PRCs: Help recoverees quit smoking | - Some coaches took tobacco treatment specialist training and currently provide smoking cessation coaching - Use evidence-based behavioral cessation skills (e.g., education on harms of tobacco, recommend FDA-approved medication, identify triggers and how to avoid triggers, gradually tapering cigs/day) - Former smoking PRCs use their lived experience with tobacco recovery to help recoverees quit (e.g., disclose their smoking status and how they quit, validate difficulty quitting) - Apply skills from 12-step program for SUDs to tobacco use to help recoverees quit tobacco - For recoverees who aren’t motivated to quit tobacco, approach topic gradually by first “planting seeds” about quitting at the right time in the conversation (e.g., not when recoveree is intoxicated) | *“We should be the conversation starters because one of the things that we, as recovery coach, we consider ourself agent of change, cheerleaders, role models. So as a recovery coach, I model. ‘Hey, I used to smoke two packs of cigarettes a day, and now I don't. And this is my story,’ and then I share the story that I shared with you this morning. And everybody's story is different, and every pathway is different.” (former smoking PRC)*  *“So you can always revisit it. I never give up. I always talk to my folks even if they're adamant about not stopping cigarette use months later. And it could be something really subtle, "Hey, have you thought about your cigarette use again?" I don't have to get critical about it and this and that, just one sentence, "Hey, have you thought about what we spoke about about tobacco?" just kind of plant those little seeds.”(former smoking PRC)* |
|  | Former smoking PRCs: PRC recommends e-cigarettes to recoverees to help them quit or reduce cigarette use++  All PRCs: PRCs don’t recommend e-cigarettes for quitting smoking as they don’t know enough information about them |  | *“I do ask people if they're interested in it [e-cigarette], if they've thought about it, if they've ever used it. The way I stopped smoking was with a vape pen, and I tapered my milligrams down, and so I just share that experience with them. That it felt easier because I was able to kind of control the amount of nicotine that was in each clip or whatever they call them. And I was just in control of that, and for some reason, it was easier for me to stop smoking that way, so I really share that.” (former smoking PRC)*  *“I know some people have had some success vaping and cutting back or cutting themselves off cigarettes and tobacco products by basically--I don't necessarily know that it's a step down, but at least that seems to be the general consensus is that it's a step down from smoking. So that's kind of how I framed it to people is, if you're not ready to quit nicotine or tobacco totally, this might be a good step down and using it as a sort of harm reduction tool.” (former smoking PRC)* |
| Pro-smoking Practices | | | |
|  | Current smoking PRCs: Accompany recoverees outside to smoke | - Taking a recoveree outdoors to smoke can help them feel more comfortable and relaxed when they appear distressed - Smoking outside together facilitates social connection and rapport between PRC and recoveree - In inpatient unit, NRT often isn’t helping enough so coaches bring recoverees outdoors to smoke | *“It’s the only deep breath they take all day… can actually slow down for the six or seven minutes that it takes to smoke a cigarette.” (current smoking PRC)*  *“So we went outside and we smoked. And as I said, sometimes that is just-- for whatever reason, is just two people talk and smoke. It's no longer in a clinical office setting. It's no longer me sitting at a desk while they're sitting in a chair. You know what I mean? It's just a couple of people talking at that point. So yes, some of the best therapy is done out there, as strange as that may sound.” (current smoking PRC)* |
|  | All PRCs: PRCs using cigarettes for harm reduction in SUD treatment (as a tool to avoid worse SUD outcomes) | - PRCs take recoverees from inpatient unit outside to avoid self-directed discharges and calm distressed/impulsive recoverees - PRCs provide recoverees with cigarettes so recoverees won’t take used cigarettes off the ground to smoke | *“Whether it's the interaction of me physically bringing them outside and doing therapeutic work, having one-on-one therapeutic work conversation, the nicotine, whatever it is, it's a harm reduction approach because they're not leaving to go use [illicit substances] to cope with the high stress of being in the hospital, right? They're not getting Ativan. The hospital's not giving them medications to cope with their anxiety because they're a user of substances. So they're not getting any type of benzo because we can't do that. You're having anxiety, "How do I get rid of that? I'm trying to deep breathe, but that's not helpful." (former smoking PRC)* |
| **What role should PRCs play in smoking cessation?** |  |  |  |
|  | Current smoking PRCs: Minimal role  Former smoking PRCs: Minimal role due to lack of training | - Current smoking PRCs should not help with smoking cessation as it would be hypocritical and a negative social model - PRCs have their hands full with other issues (e.g., other SUDs) - PRC concerns that they are not sufficiently trained in smoking cessation and thus only trained clinicians should assist recoverees with cessation | *“I think, truthfully, we have so much to do as it is with the ever-evolving landscape of drugs and alcohol. I mean, we have our hands full.” (unknown smoking status PRC***)*  *“But then I also can't be hypocritical if I'm smoking myself and then talking to them about it as well-- talking to them about quitting [smoking].” (current smoking PRC)*  *“That's not my lane. I need to stay in my lane with smoking cessation, but I can refer you, I can direct you… I would say, ‘You know what, I don't know much about it. I can give you my experience with it, but let me direct you to the people that know more about it.’”(former smoking PRC)* |
|  | All PRCs: PRC should play whatever role the patient wants the PRC to play | - PRC should be patient-centered as primary role and be guided by whatever patient’s goal is related to tobacco cessation | *“I feel like I would play the role of just meeting them where they're at. And if that's [tobacco cessation] something that they want to talk about, then I'll I'm all for talking about it. But I just won't be the one initiating the conversation about them quitting smoking.” (former smoking PRC)*  *“Well, I think it's up to the individual, right? My job isn't to get people to do things. My job is to get people to where they want to get.” (current smoking PRC)* |
|  | Former or never smoking PRCs: Large role in helping with smoking cessation | - PRCs should be a model of non-smoking for recoverees (e.g., share own experience with successful quitting) - All PRCs should all be trained in smoking cessation (e.g., complete TTS trainings) given how many recoverees smoke - Recommend FDA-approved cessation medications and can get prescriber assistance from clinic MDs as needed - Important to be patient-centered and never force cessation on any recoverees | *“Usually, I try to have a very generic conversation with somebody around, ‘Hey, I'm aware that there is [cessation] medication out there that can help you manage cravings. I'm not an addictions nurse practitioner or an addictions specialist by any means, so I can't speak to what's out there, how it works, why it works, but I can put you in touch with somebody who can have that conversation with you.’” (former smoking PRC)* |
| **Where does smoking cessation currently fall in terms of a priority to address in your work with your recoverees?** |  |  |  |
|  | Former/Never Smoking PRCs: High or very high priority | - Should be the same priority as any other substance in SUD treatment - Second most important priority in SUD treatment | *“…And I think it should be [tobacco smoking] the priority to address. But I think that due to the fact that it's normalized in society so much because it's legal and sold everywhere, it has a different look at it. It's similar to alcohol, as opposed to other illicit street drugs.” (former smoking PRC)*  *“Just as much as all the rest of the substances that folks are using illicitly. Yeah. I think it's right up there. It's a pretty high priority.” (former smoking PRC)*  *“I think it's a high priority. I just think it's not addressed much because it's just looked at as like a-- it's not anything that's causing a lot of harm. I think it does, but I don't think people really look at it like that. (never smoking PRC)”* |
|  | All PRCs: Minimal priority | - Should focus on other more pressing issues (i.e., SMI, homelessness, other substance use) that is a more immediate health threat - Hard to remember to address smoking cessation (often forget to), but agrees it is a high priority | *“I don't tell them not to, but I do say, ‘let's take care of the stuff that's going to potentially kill you sooner.’” (never smoking PRC)* |
|  | All PRCs: depends on recoverees interest in cessation | - PRCs are patient-centered and only address tobacco use if it is a recoverees goal for coaching - Offering smoking cessation to a recoveree who isn’t interested would damage rapport | *“If they are not interested in quitting smoking, it is really low [priority]. If they've expressed interest or have made comments like, "I know I should quit, but I don't know how to," or, "I know I should quit, and it's not the time," then it will be bumped up higher on the priority list because, somewhere, there's a goal there. But if the patient's not interested, then it's bottom.” (former smoking PRC)*  *“If they want to talk about it, I'm happy to do it. I don't encourage you one way or the other to smoke or not smoke.” (former smoking PRC)* |
| **How does PRC’s own use of cigarettes help or harm ability to help a recoveree quit smoking?** |  |  |  |
|  | All PRCS: Smoking status doesn’t influence ability to help+ |  | *“I don't think it would hurt or harm in any way. Because like I said, people smoke, whether you're a peer recovery coach and you smoke or a recoveree who smokes. I mean, and I don't think it harms. I mean, sometimes if you doing peer outreach and you're outside, and maybe you're the coach who's smoking. Your recoveree is sitting on the bench and you guys are smoking a cigarette. I don't think there's any judgment call there.” (former smoking PRC)* |
|  | Former smoking PRCs: Smoking status plays large role in helping recoverees quit (former smoking PRCs can and should help coach recoverees on cessation) | - Former smoking PRCs can use their own experience successfully quitting to coach others - Can help recoverees quit all SUDs at once including tobacco - Current smoking PRCs should not help recoverees quit smoking as this would be a harmful model and judgmental/hypocritical | *“I'd also provide my personal experience with the way that I quit cigarettes to be able to identify with them how hard it was, being compassionate, understanding that it's not easy, that it is a habit, and it is an addiction, and kind of looking at the way that it disrupts their overall quality of life and kind of moving towards how can we come up with a gameplan to at least lower their amount of cigarettes that they're using. What are realistic goals and realistic points to get you to the point where you don't have to depend on a cigarette anymore or nicotine use?” (former smoking PRC)*  *“I mean, for someone that starts talking about how they would like to quit smoking cigarettes, that's kind of my way of easing into that conversation and being able to share my own experience and telling them, ‘Yeah. I put cigarettes down five years ago,’ and that leads to one question after another to me giving them some resources and some ways to help them out.” (former smoking PRC)*  *“But the harm that I can think of is kind of like that, ‘Do as I say, not as I do,’ kind of thing. Kind of like if someone came to me and told me to stop smoking, and they were actively smoking, I'd kind of be like, ‘Why don't you focus on yourself? Please f*ck off.’" (former smoking PRC)* |

Note. SUD, substance use disorder; PRC, peer recovery coach. The smoking status listed in the “themes” column refers the smoking status of the coach that most often endorsed a given theme. However, for many themes, there was some endorsement/agreement across coaches all smoking statuses.

*This subject endorsed “preferred” not to answer for smoking status on the quantitative survey. On the qualitative interview they endorsed current use of smokeless tobacco (chew).

++theme only endorsed by a few coaches

+ theme was only endorsed by a few coaches (one former smoker and one never smoker)
